# Genomic Analysis of Carbapenem-Resistant *Pseudomonas aeruginosa* Isolated From Urban Rivers Confirms Spread of Clone Sequence Type 277 Carrying Broad Resistome and Virulome Beyond the Hospital

**DOI:** 10.3389/fmicb.2021.701921

**Published:** 2021-09-03

**Authors:** Fernanda Esposito, Brenda Cardoso, Herrison Fontana, Bruna Fuga, Adriana Cardenas-Arias, Quézia Moura, Danny Fuentes-Castillo, Nilton Lincopan

**Affiliations:** ^1^Department of Clinical Analysis, School of Pharmacy, University of São Paulo, São Paulo, Brazil; ^2^One Health Brazilian Resistance Project (OneBR), São Paulo, Brazil; ^3^Department of Microbiology, Institute of Biomedical Sciences, University of São Paulo, São Paulo, Brazil; ^4^Federal Institute of Education, Science and Technology of Espírito Santo, Vila Velha, Brazil; ^5^Department of Pathology, School of Veterinary Medicine and Animal Sciences, University of São Paulo, São Paulo, Brazil

**Keywords:** critical-priority pathogens, aquatic environments, carbapenemase, *Galleria mellonella*, resistome, virulome, genomic surveillance, One Health

## Abstract

The dissemination of antibiotic-resistant priority pathogens beyond hospital settings is both a public health and an environmental problem. In this regard, high-risk clones exhibiting a multidrug-resistant (MDR) or extensively drug-resistant (XDR) phenotype have shown rapid adaptation at the human-animal-environment interface. In this study, we report genomic data and the virulence potential of the carbapenemase, São Paulo metallo-β-lactamase (SPM-1)-producing *Pseudomonas aeruginosa* strains (Pa19 and Pa151) isolated from polluted urban rivers, in Brazil. Bioinformatic analysis revealed a wide resistome to clinically relevant antibiotics (carbapenems, aminoglycosides, fosfomycin, sulfonamides, phenicols, and fluoroquinolones), biocides (quaternary ammonium compounds) and heavy metals (copper), whereas the presence of exotoxin A, alginate, quorum sensing, types II, III, and IV secretion systems, colicin, and pyocin encoding virulence genes was associated with a highly virulent behavior in the *Galleria mellonella* infection model. These results confirm the spread of healthcare-associated critical-priority *P. aeruginosa* belonging to the MDR sequence type 277 (ST277) clone beyond the hospital, highlighting that the presence of these pathogens in environmental water samples can have clinical implications for humans and other animals.

## Introduction

Carbapenem-resistant *Pseudomonas aeruginosa* are a leading cause of hospital-acquired infections and have become a health priority (Tacconelli et al., 2018). Efforts have been made to prevent colonization, infection, and decrease mortality. Based on that, the WHO proposed a global priority pathogen list of multidrug-resistant (MDR) bacteria to drive research, discovery, and development of new antibiotics. Along with MDR *P. aeruginosa*, the critical pathogens WHO list included *Acinetobacter baumannii* and bacteria from Enterobacterales group (Tacconelli et al., 2018). They were categorized as critical priority through the use of multi-criteria, including being resistant to a large number of antibiotics, such as carbapenems and third generation cephalosporins, the best available options for treating MDR pathogens (Babu et al., 2020). Worryingly, carbapenem-resistant *P. aeruginosa* can cause severe and often deadly infections such as bloodstream infections, pneumonia, and osteomyelitis (Fernández-Barat et al., 2017; Pliska, 2020; Jean et al., 2020; Bobrov et al., 2021; Rosales-Reyes et al., 2021). Carbapenem resistance is usually multifactorial, including overexpression of efflux pumps (i.e., *mexAB*-*oprM*), deficiency or repression of the porin gene (*oprD*), alterations in the penicillin-binding proteins (PBPs), and chromosomal overexpression of cephalosporinase gene *ampC* (Van Nguyen et al., 2018; Gajdács, 2020; Xu et al., 2020). Moreover, resistance may be acquired by the selection of mutations in chromosomal genes or horizontal uptake of resistance determinants. However, carbapenem resistance has been most associated with production of carbapenemases, which include serine β-lactamases and metallo-β-lactamases (MβLs) (Polotto et al., 2012; Lupo et al., 2018), whereas high-risk global clones have been associated with MDR or extensively drug resistant (XDR) phenotypes. Currently, global *P. aeruginosa* high-risk clones include sequence types (STs) ST235, ST111, ST175, ST233, ST244, ST277, ST298, ST308, ST357, and ST654 (Del Barrio-Tofiño et al., 2020; Kocsis et al., 2021). Specifically, the ST277 has been sporadically reported in Asian, North American, and European countries, whereas in Brazil is highly prevalent (Gales et al., 2003; Hopkins et al., 2016; Del Barrio-Tofiño et al., 2020; Silveira et al., 2020; Kocsis et al., 2021). The success of the Brazilian endemic clone ST277 is associated with carbapenem resistance due to production of the MβL SPM-1 (Gales et al., 2003; Cipriano et al., 2007; da Fonseca et al., 2010; Nascimento et al., 2016; Silveira et al., 2020). Worryingly, SPM-1-producing *P. aeruginosa* have been identified in hospital sewage and hospital wastewater treatment plants (Fuentefria et al., 2009; Miranda et al., 2015), denoting potential to spread throughout the aquatic environment, enabling human exposure and transmission. However, although whole genome sequencing (WGS) of human SPM-1-positive isolates have been performed (Nascimento et al., 2016; Galetti et al., 2019), sequence data from environmental isolates have not been provided for comparative genomic studies. Based on WHO list priority pathogens criteria, which included pathogen mortality, hospital and environment transmissibility and limited treatment options, recognition and genomic characterization of critical priority pathogens is an essential first step to understanding their dynamic of acquisition/dissemination and ultimately to development of preventive intervention strategies (Hendriksen et al., 2019). In this study, we report genomic data and the virulence potential of carbapenem-resistant SPM-1-positive *P. aeruginosa* strains isolated from polluted urban rivers, in Brazil.

## Materials and Methods

### *Pseudomonas aeruginosa* Strains and Antimicrobial Susceptibility Profiles

During a Brazilian surveillance study (OneBR project) conducted to investigate the burden of antimicrobial resistance in impacted aquatic environments, two *P. aeruginosa* strains [Pa19 (ONE609) and Pa151 (ONE610)] were isolated from two different locations along the Tietê (TIET-04900; S 23° 31' 18'', W 46° 37' 52'', S 23° 27' 16'', and W 46° 54' 36'') and Pinheiros (PINH-04900; S 23° 31' 52'' and W 46° 44' 54'') Rivers in São Paulo, Brazil (Turano et al., 2016). Tietê River stretches through São Paulo state from east to west for approximately 1,100km, while Pinheiros River is a tributary of the Tietê River that runs 25km across the city. In this study, both strains were subjected to WGS for investigation and comparative genomic studies using five public sequences from nosocomial SPM-1-positive *P. aeruginosa* strains, previous reported (Silveira et al., 2014, 2020; Nascimento et al., 2016; Galetti et al., 2019). Susceptibility profiles were investigated by disk-diffusion method (CLSI, 2021).

### Whole Genome Sequencing and Genomic Analysis

Genomic DNA of Pa19 and Pa151 were extracted using PureLink Quick Gel Extraction & PCR Purification Combo Kit (Life Technologies, Carlsbad, CA). The Illumina paired-end libraries were constructed using a Nextera XT DNA Library Preparation Kit (Illumina Inc.), according to the manufacturer’s guidelines. Whole genome sequencing was performed using an Illumina MiSeq platform with 300-bp read lengths. Reads were *de novo* assembled using SPAdes 3.13,^1^ and the resulting contigs were automatically annotated by NCBI Prokaryotic Genome Annotation Pipeline (PGAP) version 3.2.^2^ Antibiotic resistance genes were predicted using ResFinder 4.1^3^ and the Comprehensive Antibiotic Resistance Database (CARD).^4^ Multi-locus Sequence Typing prediction was performed using MLST v.2.0.^5^ Heavy metal (HM) resistance genes were manually identified using the NCBI database^6^ and Geneious Prime version 2020.04 (Biomatters, New Zealand). Additionally, phage prediction was performed by Genome Detective Virus Tool software.^7^ The *rmtD* gene was detected and aligned by BLASTn (Alikhan et al., 2011) against the *rmtD1* allele of the *P. aeruginosa* (PA0905 strain), recovered from a human patient (GenBank accession number. DQ914960). Genetic context analysis of *bla*_SPM-1_ and *rmtD1* resistance genes of Pa151 were performed with BLASTn algorithm and manually curated using Geneious Prime version 2020.04 (Biomatters, New Zealand).

Moreover, virulence genes, efflux systems, and regulators were determined through the Virulence Factor Database.^8^ Serotype was predicted using Past 1.0.^9^ SNP-based phylogenetic analysis was performed by using Prokka 1.13.4^10^ for pangenome annotation, followed by Roary 3.13.0^11^ for core genome analysis. SNP-sites tool^12^ was used for SNPs extraction from the core gene alignment; whereas RAxML-NG version 0.9.0^13^ for phylogenetic construction and a maximum likelihood tree based on SNP alignment. Additionally, comparative genomic analysis of *P. aeruginosa* sequences was performed by BRIG v.0.95 using the BLASTn algorithm and Island viewer 4.0.

All genomic analysis were based on comparison of sequences of environmental Pa151 (Pinheiros River, GenBank accession number: PHSS00000000.1) and Pa19 (Tietê River, GenBank accession number: PHST01000000) strains, against publically available genome sequences (data obtained by using 300 bp paired-end MiSeq sequencing) of clinical SPM-1-producing *P. aeruginosa* CCBH4851 (catheter tip, GenBank accession number CP021380.2), PA1088 (urine, GenBank accession number CP015001.1), PA11803 (bloodstream, GenBank accession number: CP015003.1), PA12117 (bloodstream, GenBank accession number: LVXB00000000.1) and PA7790 (tracheal aspirate, GenBank accession number: CP014999.1) strains, which were retrieved from NCBI GenBank database.^14^ For SNP-based analysis, the genome of the *P. aeruginosa* strain PAO1 (ST549) was used as reference (GenBank accession number: AE004091.2).

### Virulence Potential of Carbapenem-Resistant *P. aeruginosa* Strains in the *Galleria mellonella* Larvae Model

The virulence potential of *P. aeruginosa* Pa19 and Pa151 strains was evaluated using the *Galleria mellonella* infection model (Tsai et al., 2016). In brief, groups of *G. mellonella* containing 10 larvae of nearly 0.25–0.35g (supplied by the Institute of Biomedical Sciences of the University of São Paulo, Brazil) were infected with 10^4^CFU/ml of each strain per larvae, by injecting a 10μl aliquot in PBS, into the body of the larvae *via* the last left proleg, using a sterile ultra-fine needle syringe (Fuentes-Castillo et al., 2019). Survival was monitored every hour, for 96h. Two biological replicates and two experimental replicates were performed with a group of 10 larvae per strain, in each replicate. SPM-1-producing *P. aeruginosa* clinical strain PA1088 was used as comparative control (Toleman et al., 2002). Moreover, a control group inoculated with sterile PBS was used in each biological and experimental replication assay, in order to verify that the larvae would not be killed by physical trauma. Survival curves were plotted using the Kaplan-Meier method, whereas statistical analyses were performed by the log rank test with *p* <0.05 indicating statistical significance (OriginLab Software, Northampton, Massachusetts, United States).

## Results

**Figure 1:**
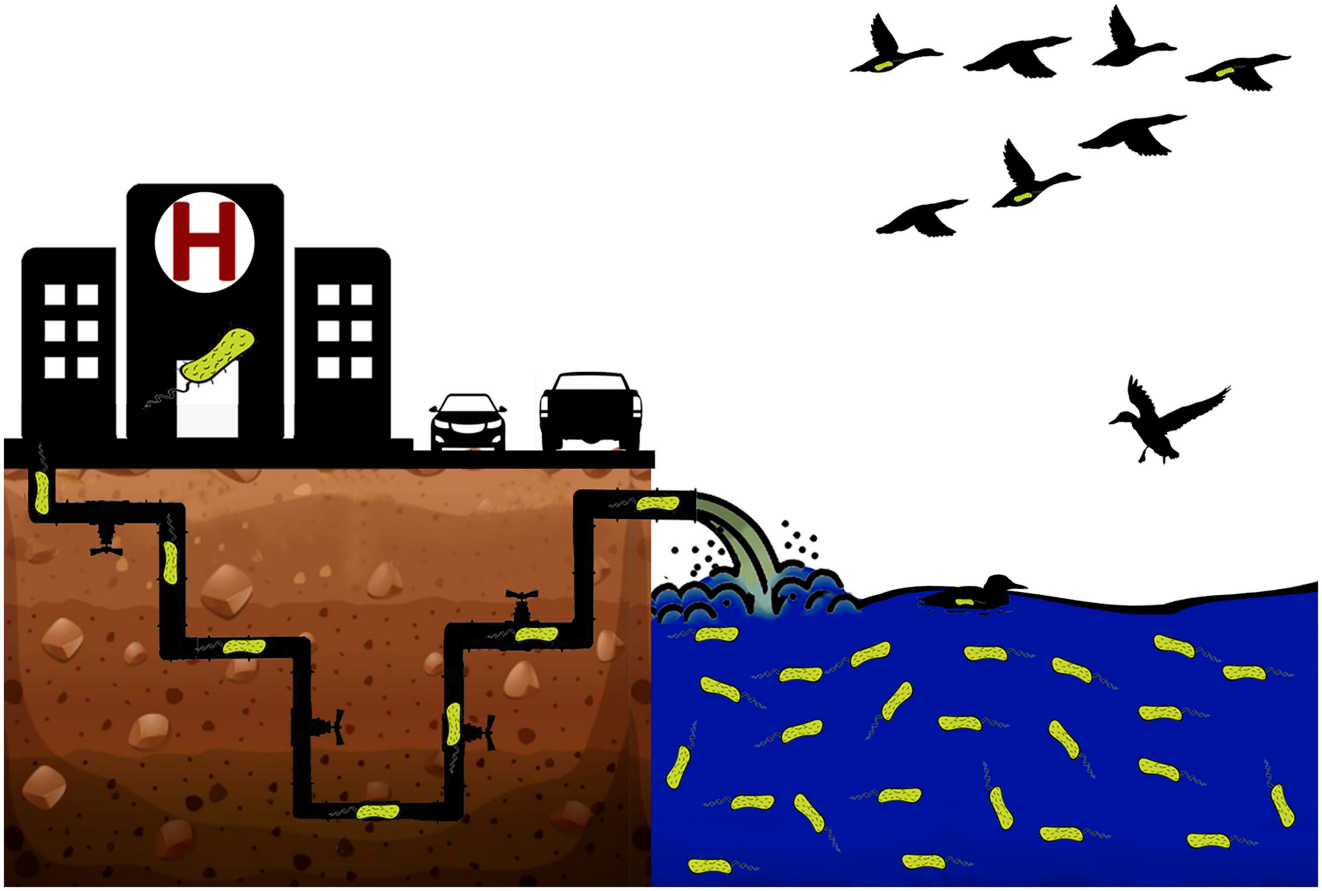
Schematic representation of hypothesis proposed for spread of carbapenemase (SPM-1)-producing *Pseudomonas aeruginosa* clone ST277 beyond the hospital, in Brazil, based on genomic data analyzed in this study.

In this study, two carbapenemase (SPM-1)-producing *P. aeruginosa* ST277 (Pa19 and Pa151 strains) isolated from impacted urban rivers in São Paulo, Brazil, were sequenced. As this clone has been endemic in Brazilian hospitals, being also identified in migratory birds (Figure 1), we have additionally performed a comparative analysis with publically available genomes obtained from ST277 lineages from human infections.

Genome sequencing yielded a total of 968,818 and 473,825 paired-end reads assembled into 395 and 337 contigs, with 305 and 299x of coverage, to Pa19 and Pa151 strains, respectively. The genome size of Pa19 was calculated at 6,927,007bp, with a GC content of 67.8%, comprising 6,956 total genes, 60 tRNAs, three rRNAs, four ncRNAs, and 155 pseudogenes (accession number: PHST00000000.1). On the other hand, genome size of Pa151 was calculated at 6,799,801bp, with a GC content of 66.9%, comprising 6,747 total genes, 59 tRNAs, three rRNAs, four ncRNAs, and 123 pseudogenes (accession number: PHSS00000000.1). Genomic information of *P. aeruginosa* Pa19 and Pa151 strains are available on the OneBR platform^15^ under ONE609 and ONE610 ID numbers, respectively.

**Figure 2:**
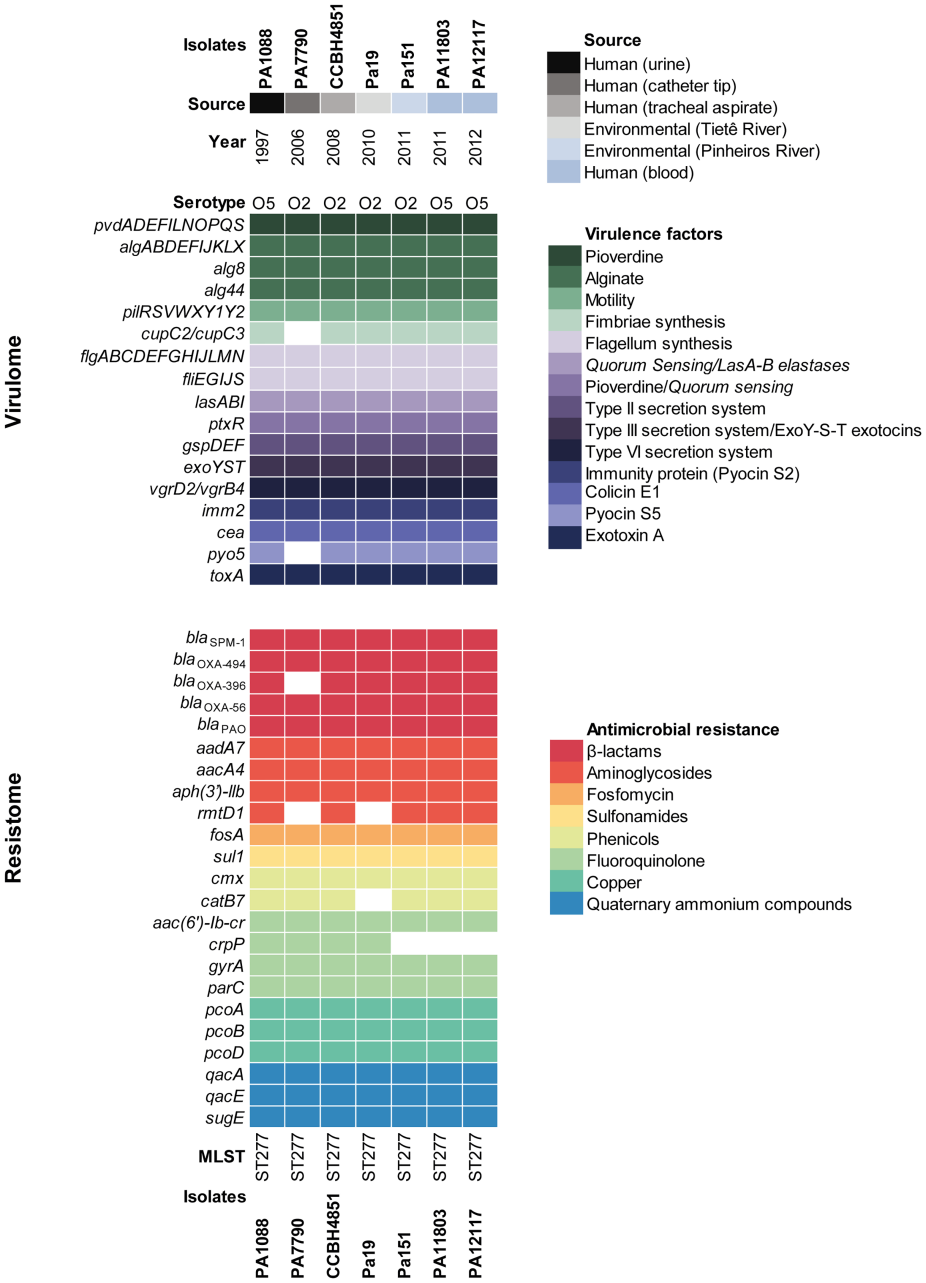
Heatmap showing the distribution of virulence and resistance genes in environmental and clinical SPM-1-positive *P. aeruginosa* strains of ST277 clone. Virulence genes are listed along with their functions and demarcated by colored squares. Resistome is demarcated by colored squares under genes names grouped by their antimicrobial resistance classes. *Pseudomonas aeruginosa* strains are indicated by colors displaying their source and year of isolation.

Environmental Pa19 and Pa151 strains displayed a MDR profile to ticarcillin-clavulanate, cefepime, ceftazidime, imipenem, meropenem, amikacin, gentamicin, nalidixic acid, ciprofloxacin, levofloxacin, and trimethoprim-sulfamethoxazole, and genomic analysis revealed a wide resistome to β-lactams (*bla*_SPM-1_, *bla*_OXA-56_, *bla*_OXA-396_, *bla*_OXA-494_, and *bla*_PAO_), aminoglycosides [*aacA4*, *aadA7* and *aph(3')-llb*], fluoroquinolones [*aac(6')lb-cr*, and *gyrA* (T83I) and *parC* (S87L) point mutations], phenicols (*cmx*), sulphonamides (*sul1*), and fosfomycin (*fosA*), which was predicted in agreement with the phenotype. Additionally, Pa151 strain harbored the *rmtD1* and *catB7* genes related to aminoglycosides and chloramphenicol resistance, respectively (Figure 2). On the other hand, the *crpP* gene associated with fluoroquinolone resistance, was only identified in the Pa19 genome. Genes associated with resistance to heavy metal [copper (*pcoABD*)], and quaternary ammonium compounds (*qacE*, *qacA*, and *sugE*) were also identified in both environmental *P. aeruginosa* strains (Figure 2).

**Figure 3:**
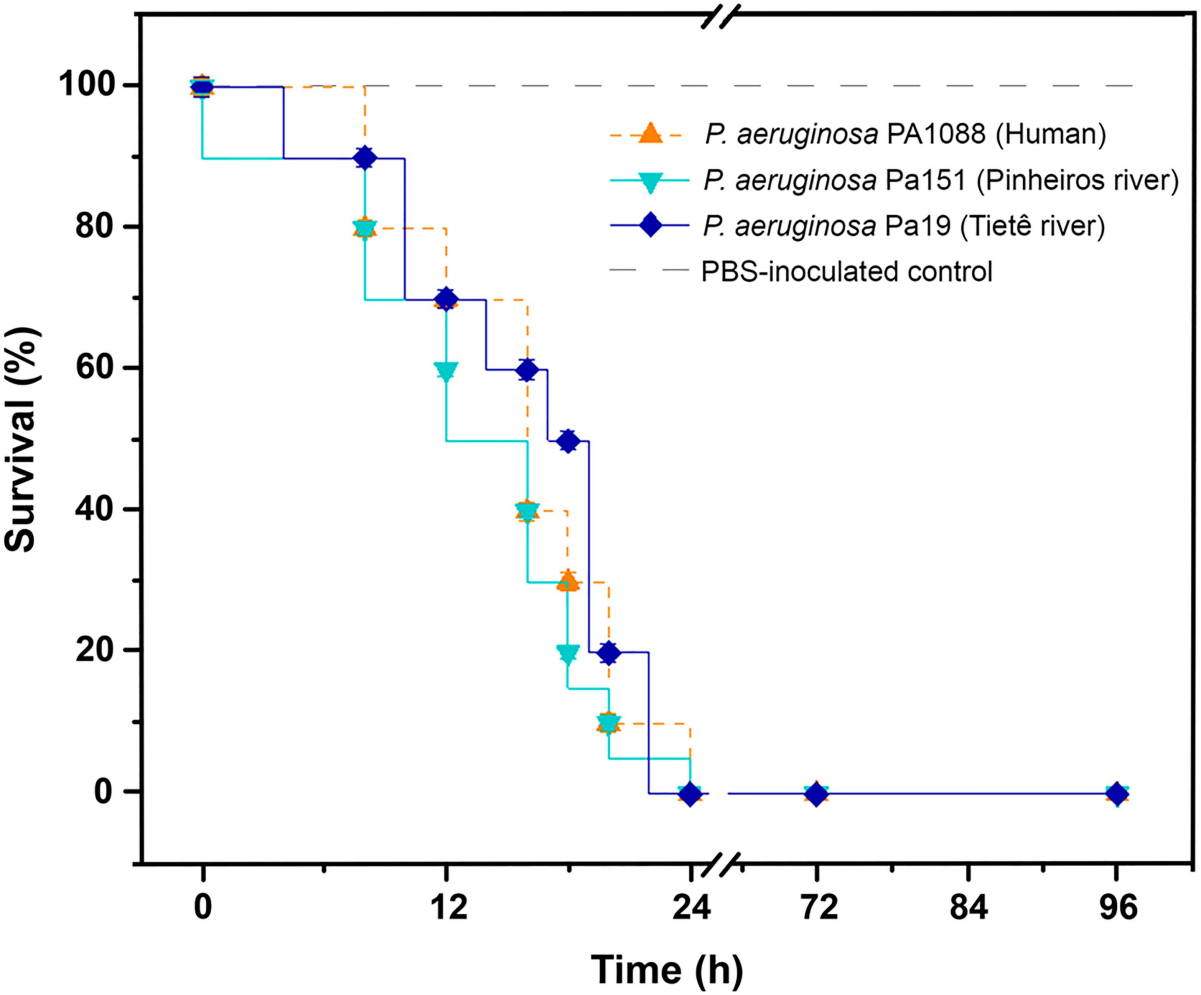
Virulent behavior of SPM-1-producing *P. aeruginosa* isolates. Kaplan-Meier survival curves of *Galleria mellonella* infected with 10^4^CFU/larva of *P. aeruginosa* Pa19 strain (dark-blue line), *P. aeruginosa* Pa151 strain (blue line), and *P. aeruginosa* PA1088 (orange line). Environmental Pa19 and Pa151 strains, and the clinical PA1088 strain killed 100% of larvae at 24h post-infection. PBS-inoculated control group (light-gray dashed line) presented 100% of survival. For each strain, groups containing 10 *G. mellonella* larvae in each replicate were evaluated in two biological and experimental independent assays.

Virulome analysis of environmental Pa19 and Pa151 revealed a wide virulome. In fact, both lineages carried the *quorum sensing* (*lasA*, *lasB*, *lasI*, and *ptxR*), alginate (*alg* cluster), siderophore production (*pvdA*, *pvdF*, and *pvdG*), fimbriae (*cup* family), flagellum (*flgABCDEFGHIJLMN*) synthesis, immunity protein (*pyo5*, *imm2*), colicin (*cea*), types II (*gspDEF*), III (*exoYST*), and IV (*vgrD2*/*vgrD4*) secretion systems and exotoxin A (*toxA*) genes; whereas the O2 serotype was identified in both Pa19 and Pa151 environmental strains (Figure 2). In this regard, *in vivo* experiments using *G. mellonella* larvae showed that both Pa19 and Pa151 strains killed 100% of the larvae at 24h post-infection, similarly to what was observed with the clinical SPM-1-producing *P. aeruginosa* PA1088 strain isolated from a case of urinary tract infection (Figure 3).

**Figure 4:**
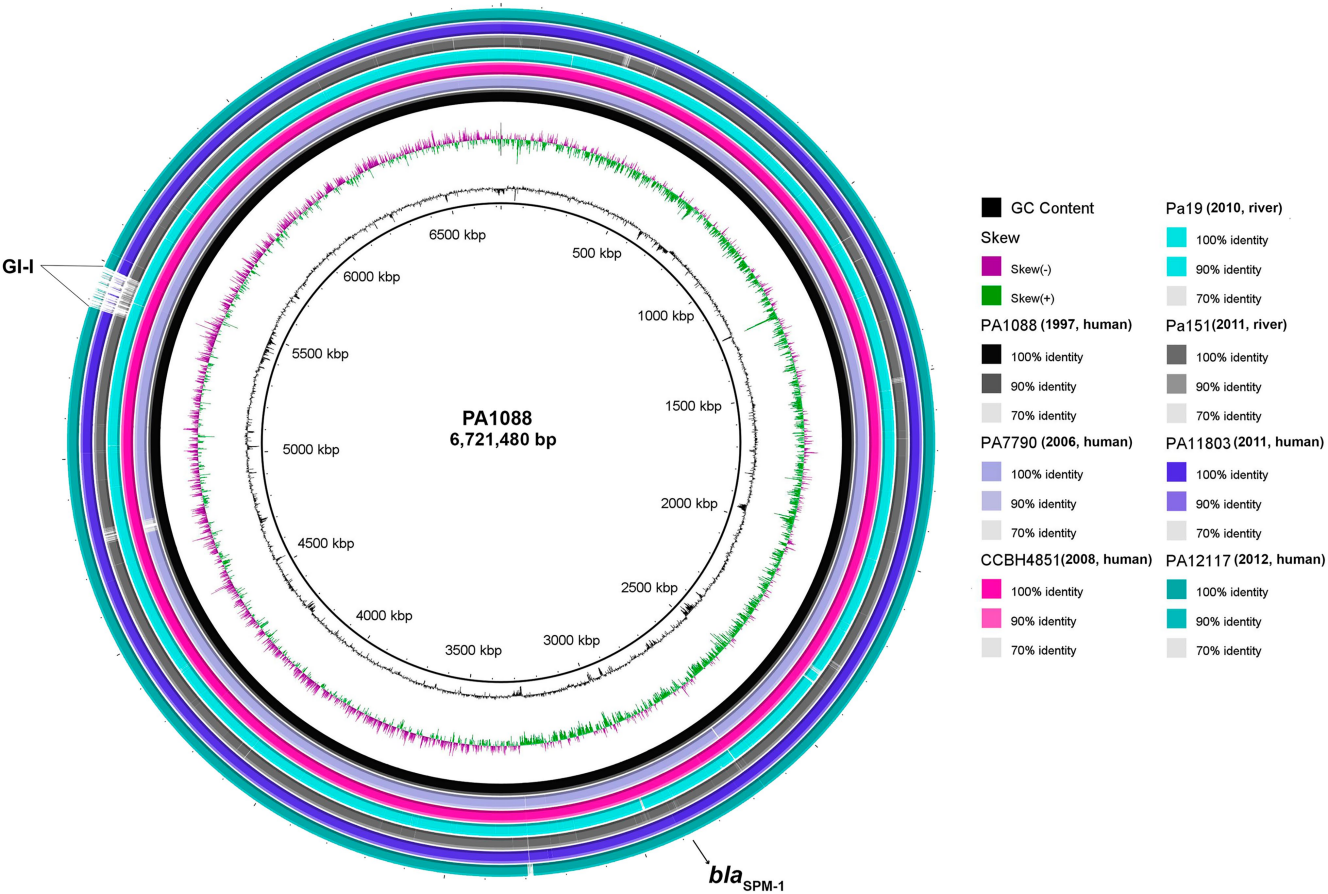
Circular genome maps of SPM-1-producing *P. aeruginosa* belonging to ST277. Circular maps were built by BLAST Ring Image Generator (BRIG) using seven *P. aeruginosa* genomes. All genomes were represented as individual rings and compared against the reference genome PA1088 (GenBank accession number: CP015001.1). The *bla*_SPM-1_ gene is indicated by a black arrow. Furthermore, several genes associated with DNA replication/repair/regulatory/defense and membrane proteins were identified in the major genomic island, indicated as GI-I.

Overall comparison of five human and two environmental ST277 genomes using BLAST Ring Image Generator (BRIG) revealed high nucleotide sequence similarities among *P. aeruginosa* strains, even for aquatic isolates recovered at least 13years after the first clinical isolate (Toleman et al., 2002). Furthermore, SNP-based phylogenetic analysis revealed that both Pa19 and Pa151 environmental strains were closely related (>94% identity) to all human SPM-1-producing *P. aeruginosa* isolates (Supplementary Table S1). However, missing regions at position 5.5Mbp, named as GI-I, in environmental Pa151 and clinical PA11803 and PA12117 genomes, were identified (Figure 4). In this regard, we observed genes encoding the following proteins: integrating conjugative element protein (*pill*), type II secretion system protein, replicative DNA helicase (*dnaB*), nucleoid-associated protein YejK (*yejK*), NADH dehydrogenase (*ndh*), cell division protein ZapE (*zapE*), ParA family protein (*parA*), plasmid stabilization protein ParE (*parE*), integrating conjugative element protein, DNA topoisomerase I (*topA*), pyocin S5 (*pyoS5*), TetR family transcriptional regulator (*tetR*), conjugal transfer protein TraG (*traG*), regulatory protein GemA (*gemA*), conjugative coupling factor TraD (*traD*), his-Xaa-Ser repeat protein HxsA (*hxsA*), his-Xaa-Ser system radical SAM maturase HxsB (*hxsB*), his-Xaa-Ser system radical SAM maturase HxsC (*hxsC*), his-Xaa-Ser system protein HxsD (*hxsD*), chaperone protein ClpB (*clpB*), and genes encoding for membrane proteins, transcriptional regulator, CRISPR-associated proteins, type II secretion system protein, phage tail sheath subtilisin-like, tail fiber protein, phage tail tape measure protein, and phage head morphogenesis protein.

**Figure 5:**
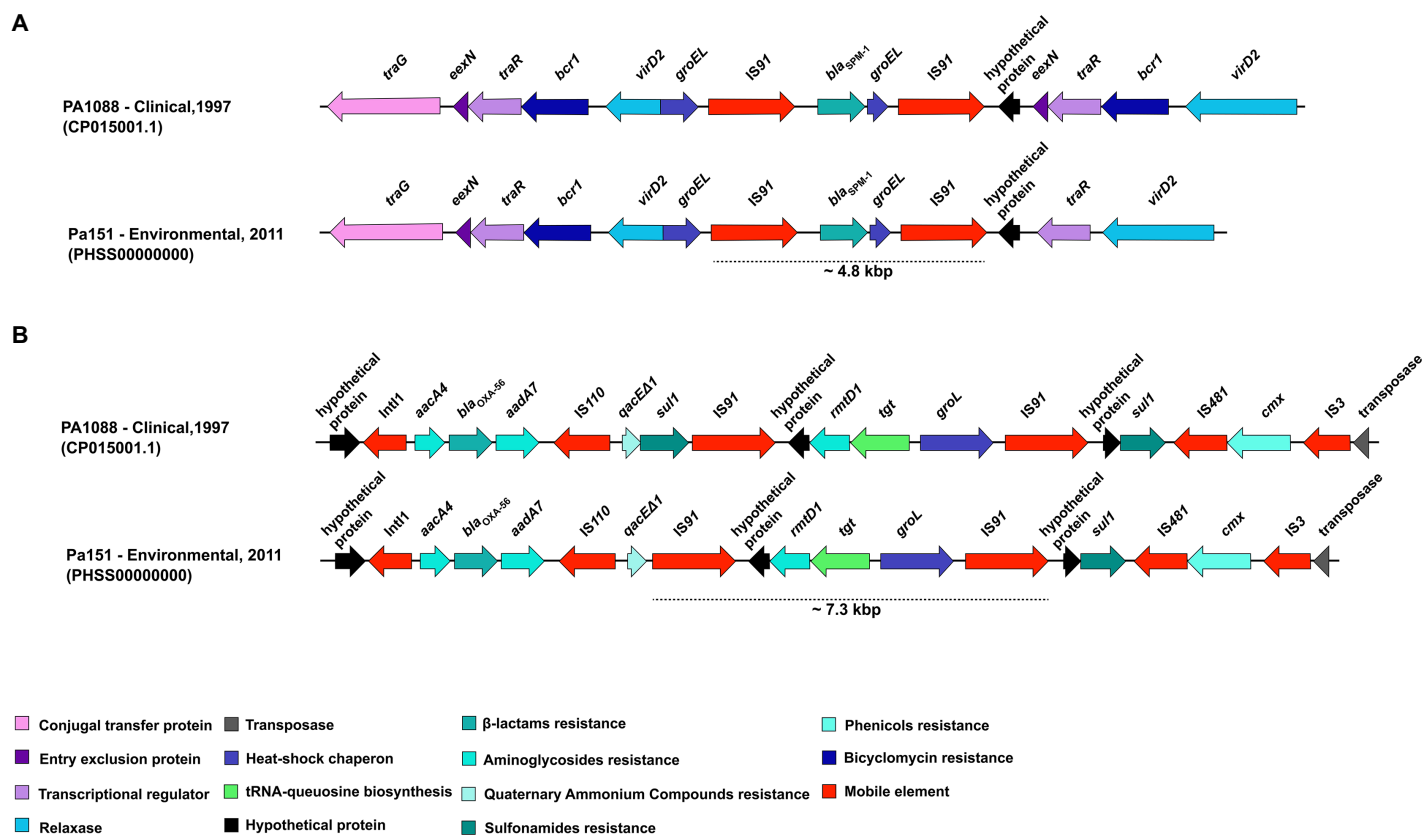
Overall comparison of genetic context of *bla*_SPM-1_ and *rmtD1* genes carried by clinical and environmental *P. aeruginosa* strains belonging to ST277. **(A)** The *bla*_SPM-1_ was flanked by a ~4.8kbp region composed of IS*91*-*bla*_SPM-1_-*groEL*-IS*91*. **(B)** The *rmtD1* was flanked by a ~7.3kbp region composed of the IS*91*-*rmtD1*-*tgt*-*groEL*-IS*91*. In addition, *aacA4*, *aadA7*, *bla*_OXA-56_, *qacEΔ1*, *sul1,* and *cmx* resistance genes were also identified along with genes encoding for hypothetical proteins, transposase; as well as IS*110*, IS*481*, and IS*3* mobile genetics elements.

Schematic representations of the genetic context surrounding *bla*_SPM-1_ genes in the environmental *P. aeruginosa* PA151 strain is presented in Figure 5A. The *bla*_SPM-1_ was flanked by a ~4.8kbp region composed of the IS*91*-*bla*_SPM-1_-*groEL*-IS*91* array. The presence of IS elements is related to horizontal gene transfer, whereas the *groEL* encodes for a heat-shock chaperon. Additionally, we also detected the *traG* (encoding a conjugal transfer protein), *eexN* (encoding the entry exclusion protein), *traR* (transcriptional regulator), *bcr1* (bicyclomycin resistance), *virD2* (gene encoding a relaxase), and hypothetical proteins. In Figure 5B is presented the genetic context surrounding *rmtD1* gene in PA151 strain. The *rmtD1* was flanked by a ~7.3kbp region composed of the IS*91*-*rmtD1-tgt*-*groEL*-IS*91* array. In addition, *aacA4*, *bla*_OXA-56_, *aadA7*, and *qacEΔ1* genes were located on a class 1 integron. Moreover, *cmx* and *sul1* resistance genes, that encodes for chloramphenicol and sulphonamide resistance, respectively, were also identified along with genes encoding hypothetical proteins, transposase, IS*110*, IS*481*, and IS*3* mobile elements.

## Discussion

Carbapenem-resistant *P. aeruginosa* are critical-priority pathogens associated with high mortality and morbidity (Georgescu et al., 2016; Tacconelli et al., 2018; Pang et al., 2019). In this regard, one of the major concerns has been the successful expansion and rapid spread of high-risk clones. In Brazil, the metallo-β-lactamase (SPM-1)-producing *P. aeruginosa* ST277 clone has gained significant attention, due to its endemicity status and further identification in migratory birds and polluted environments (Gales et al., 2003; Nascimento et al., 2016; Turano et al., 2016; Martins et al., 2018).

Worryingly, previous studies have also reported the occurrence of carbapenemase (KPC-2)-producing *Klebsiella pneumoniae* belonging to the clonal group CG258 and OXA-23-positive *A. baumannii* ST79 in the Tietê River (Oliveira et al., 2014; Turano et al., 2016), supporting an anthropogenic trend, most likely due to hospital wastewater discharge and domestic wastewaters effluents (Nascimento et al., 2017; Bartley et al., 2019; Böger et al., 2021; Popa et al., 2021). Therefore, aquatic environment could play an important role in the widespread of critical pathogens (Devarajan et al., 2017). In fact, polluted rivers could be contributing for colonization of local and migratory fauna (Martins et al., 2018; Narciso et al., 2020).

In order to elucidate the genomic aspects associate with the environmental dissemination of healthcare-associated *P. aeruginosa* ST277, we performed a comparative genomic analysis, extracting clinically relevant information (i.e., resistome, virulome, and phylogenomic). Interestingly, although the strains were isolated in different years (1997–2012), we observed that clinical and environmental SPM-1-producing *P. aeruginosa* strains share a common resistome and virulome.

Although, oral antibiotics have been successfully used in the treatment of bacterial infection, for *P. aeruginosa* few therapeutic options are available, being restricted to some fluoroquinolones, including ciprofloxacin, levofloxacin, and prulifloxacin, which are given alone or in combinations with a second intravenously or inhaled anti-pseudomonal antibiotic such as β-lactams (piperacillin/tazobactam, ceftolozane/tazobactam, ceftazidime, cefepime, or carbapenems) and/or aminoglycosides (tobramycin, amikacin, or gentamicin) (Tümmler, 2019; Ibrahim et al., 2020; Nisly et al., 2020). However, under a clinical perspective, even co-resistance to carbapenems and aminoglycosides in ST277 have already been reported and limited therapeutic options. This resistance profile is mediated by *bla*_SPM-1_ and *rmtD* genes, respectively (Doi et al., 2007). Strikingly, in some ST277, including environmental (Pa19) and human (PA7790) lineages, the *rmtD* gene was not found. On the other hand, the *rmtD1* identified in the environmental Pa151 strain, displayed 100% identity to the *rmtD1* gene from *P. aeruginosa* PA0905 strain, recovered from a human patient in 2005, in Brazil (Doi et al., 2007). The *rmtD1* was subsequently identified in *K. pneumoniae* and other Enterobacterales in Latin America, Europe, and North America (Bueno et al., 2016). Since acquisition of this gene has been linked to transposition events (Doi et al., 2007; Nascimento et al., 2016), most likely genomic plasticity of *P. aeruginosa* has led to the dissemination of *rmtD*^+^ and *rmtD*-ST277 lineages (Silveira et al., 2020). In Brazil, occurrence of *rmtD* has also been documented in *Escherichia coli* and *K. pneumoniae* (Yamane et al., 2008; Leigue et al., 2015).

The *bla*_SPM-1_, IS*91*-*bla*_SPM-1_-*groEL*-IS*91* gene array has been previously identified within a Tn*4371*-like integrative and conjugative element (ICE_Tn4371_6061) considered stable in the chromosome loci of *P. aeruginosa* ST277 strains recovered from humans and animals (Fonseca et al., 2015; Nascimento et al., 2016). Since ICEs are genetic mobile platforms that play an important role during bacterial evolution, they are overlooked as vectors in the spread and resistance emergence in many bacterial species (Fonseca and Vicente, 2016). Moreover, the genetic context of *rmtD1* (IS*91*-*rmtD1-tgt*-*groEL*-IS*91*) identified in the environmental strain was similar to previous descriptions, where the presence of the *rmtD* gene in clinical isolates was associated to the Tn*As3* transposon (Fonseca et al., 2015; Nascimento et al., 2016).

In human and aquatic *P. aeruginosa* ST277 isolates the resistome was not restricted to antibiotics, and the presence of genes conferring tolerance to copper and QAC biocides was further detected. Currently, there is a growing concern about biocides that pollute aquatic environments, especially QACs, since these compounds are widely used in domiciliary and hospital settings, as disinfectants, soaps, toothpastes, and mouthwash formulations (Zubris et al., 2017; Fuentes-Castillo et al., 2020). Consequently, ecosystems impacted by HM and biocides could favor the selection and persistence of high-risk clones harboring a broad resistome (Baker-Austin et al., 2006; Zhao et al., 2012; Kim et al., 2018).

Although a limitation of this study was the lack of a known highly virulent *P. aeruginosa* to be used as a positive control in the *in vivo* assay; we observed that the virulent behavior of environmental strains was identical to clinical strains. Indeed, a wide virulome was also predicted in human and environmental *P. aeruginosa* ST277 lineages, denoting a pathogenic potential, as demonstrated in the *G. mellonella* infection model. Lipopolysaccharide (LPS) O-antigen, type IV pili, and flagella are components of the external cell wall structure of *P. aeruginosa* and play important roles in the early stage of colonization, persistence, and bacterial pathogenesis (Hauser, 2011; Behzadi et al., 2021). Furthermore, O-antigen is an important virulence factor in *P. aeruginosa* used for the detection of MDR/XDR high-risk clones (Del Barrio-Tofiño et al., 2019). Strikingly, among clinical strains were identified the serotypes O5 and O2. The latter was also identified among environment strains. Both serotypes have been associated with acute and chronic infections (Lu et al., 2014; Li et al., 2018).

Type secretion systems (TSSs) are mechanisms by which bacteria translocate a set of toxins into the cytosol of host cells and/or to the extracellular medium (Abby et al., 2016). *Pseudomonas aeruginosa* is known to have five TSSs, of which Types I (T1SS), II (T2SS), and III (T3SS) are involved in the virulence of this pathogen. Several studies have linked these TSSs with poor outcomes of patients with acute respiratory diseases (i.e., pneumonia), with T3SS being one of the most clinically relevant virulence determinants (Hauser, 2011; McMackin et al., 2019; Sarges et al., 2020). In this context, we detected ExoTSY exotoxins-encoding genes in both clinical and environmental strains. ExoTSY exotoxins are secreted by T3SS and reported to be involved in lung injury, pulmonary-vascular barrier disruption, and end-organ dysfunction in chronic infections, mainly in CF patients; as well as with mortality in animal models (Lu et al., 2014; Sarges et al., 2020; Jurado-Martín et al., 2021). Interestingly, the *toxA* gene (exotoxin A), which is present in the most clinically *P. aeruginosa* strains (Khosravi et al., 2016) was also identified in environmental strains. Exotoxin A has been associated with tissue damage related to poor outcomes of burn patients (Khosravi et al., 2016). In fact, the broad virulome harbored by *P. aeruginosa* ST277 seems to be associated with a remarkable ability to adapt to different human and non-human conditions (Jurado-Martín et al., 2021).

In brief, from comparative analysis, our data revealed that Pa19 and Pa151 environmental strains presented slight variations when compared against clinical strains, suggesting a high degree of genetic conservation, regardless isolation data and exposition to contaminants (antibiotics and biocides residues) present in the polluted aquatic environments.

## Conclusion

In summary, we report genomic comparative data of antimicrobial-resistant *P. aeruginosa* isolated from aquatic environments in Brazil. The presence of SPM-1-producing *P. aeruginosa* ST277 in urban rivers could be associated with hospital effluents, since SNP-based phylogenomics showed high nucleotide sequence similarity between clinical and environmental genomes. Additionally, wide resistome and virulome have been conserved in environmental isolates, denoting that critical priority *P. aeruginosa* of the high-risk ST277 has successfully expanded beyond the hospital. Therefore, genomic surveillance is essential to rapidly identify and prevent the spread of WHO critical priority clones with One Health implications.

## Data Availability Statement

The datasets presented in this study can be found in online repositories. The names of the repository/repositories and accession number(s) can be found in the article/Supplementary Material.

## Author Contributions

FE, BC, HF, and BF performed the data analysis. FE, BC, QM, AC-A, and DF-C conducted the experiments. NL supervised the experiments and designed and coordinated the project. FE, BC, and NL wrote, reviewed, and edited the manuscript. All authors contributed to the article and approved the submitted version.

## Conflict of Interest

The authors declare that the research was conducted in the absence of any commercial or financial relationships that could be construed as a potential conflict of interest.

The reviewer AB declared a shared affiliation with no collaboration with several of the authors FE, HF, BF, and NL to the handling editor at the time of the review.

## Publisher’s Note

All claims expressed in this article are solely those of the authors and do not necessarily represent those of their affiliated organizations, or those of the publisher, the editors and the reviewers. Any product that may be evaluated in this article, or claim that may be made by its manufacturer, is not guaranteed or endorsed by the publisher.

## Footnotes

^1^ https://github.com/ablab/spades

^2^ https://www.ncbi.nlm.nih.gov/genome/annotation_prok/

^3^ https://cge.cbs.dtu.dk/services/ResFinder

^4^ https://card.mcmaster.ca/analyze/rgi

^5^ https://cge.cbs.dtu.dk/services/MLST/

^6^ https://www.ncbi.nlm.nih.gov/Traces/wgs/

^7^ https://www.genomedetective.com/app/typingtool/virus/

^8^ http://www.mgc.ac.cn/VFs/

^9^ https://cge.cbs.dtu.dk/services/PAst/

^10^ https://github.com/tseemann/prokka

^11^ https://github.com/sanger-pathogens/Roary

^12^ https://github.com/sanger-pathogens/snp-sites

^13^ https://github.com/stamatak/standard-RAxML

^14^ https://www.ncbi.nlm.nih.gov

^15^ http://onehealthbr.com/
